# Investigations into photoreceptor energy metabolism during experimental retinal detachment

**DOI:** 10.3389/fncel.2022.1036834

**Published:** 2022-11-18

**Authors:** Glyn Chidlow, Weng Onn Chan, John P. M. Wood, Robert J. Casson

**Affiliations:** Ophthalmic Research Laboratories, Discipline of Ophthalmology and Visual Sciences, University of Adelaide, Adelaide, SA, Australia

**Keywords:** retina, retinal detachment, retinal metabolism, bioenergetics, cone photoreceptor, immunohistochemistry, pyruvate

## Abstract

Retinal detachment is a sight-threatening disorder, which occurs when the photoreceptors are separated from their vascular supply. The aim of the present study was to shed light on photoreceptor energy metabolism during experimental detachment in rats. Retinal detachment was induced in the eyes of rats *via* subretinal injection of sodium hyaluronate. Initially, we investigated whether detachment caused hypoxia within photoreceptors, as evaluated by the exogenous and endogenous biomarkers pimonidazole and HIF-1α, as well as by qPCR analysis of HIF target genes. The results showed no unequivocal staining for pimonidazole or HIF-1α within any detached retina, nor upregulation of HIF target genes, suggesting that any reduction in pO_2_ is of insufficient magnitude to produce hypoxia-induced covalent protein adducts or HIF-1α stabilisation. Subsequently, we analysed expression of cellular bioenergetic enzymes in photoreceptors during detachment. We documented loss of mitochondrial, and downregulation of glycolytic enzymes during detachment, indicating that photoreceptors have reduced energetic requirements and/or capacity. Given that detachment did not cause widespread hypoxia, but did result in downregulated expression of bioenergetic enzymes, we hypothesised that substrate insufficiency may be critical in terms of pathogenesis, and that boosting metabolic inputs may preserve photoreceptor bioenergetic production and, protect against their degeneration. Thus, we tested whether supplementation with the bioavailable energy substrate pyruvate mitigated rod and cone injury and degeneration. Despite protecting photoreceptors in culture from nutrient deprivation, pyruvate failed to protect against apoptotic death of rods, loss of cone opsins, and loss of inner segment mitochondria, *in situ*, when evaluated at 3 days after detachment. The regimen was also ineffective against cumulative photoreceptor deconstruction and degeneration when evaluated after 4 weeks. Retinal metabolism, particularly the bioenergetic profiles and pathological responses of the various cellular subtypes still presents a considerable knowledge gap that has important clinical consequences. While our data do not support the use of pyruvate supplementation as a means of protecting detached photoreceptors, they do provide a foundation and motivation for future research in this area.

## Introduction

Retinal detachment, defined as the separation of the neurosensory retina from the underlying retinal pigment epithelium (RPE), is one of the most common vision-threatening conditions. The inner layers of the retina, which have a blood supply derived from the central retinal artery are not adversely impacted by retinal detachment. In contrast, rod and cone photoreceptors, which have relentless energy demands, are profoundly affected by ongoing detachment since their nutrient and oxygen supplies derive almost entirely from the choriocapillaris, which lies posterior to the RPE. Studies using feline and rodent models have demonstrated that following persistent detachment of the retina, there occurs a progressive loss of rod photoreceptors (Erickson et al., 1983; Cook et al., 1995; Hisatomi et al., 2002; Chong et al., 2008). Cone photoreceptors appear more resilient to detachment than rods, but nevertheless undergo a degenerative process that has been termed “deconstruction” (Mervin et al., 1999), which encompasses structural remodelling and prolonged downregulation of gene products (Linberg et al., 2001; Rex et al., 2002a,b; Chidlow et al., 2022), leading to functional abnormality. Although retinal reattachment surgery is routinely performed for rhegmatogenous retinal detachment, vision often does not return to normal. This is particularly relevant in individuals in which detachment involves the cone-dominant macula. Studies have shown that less than 50% of such individuals reach a final vision of 20/40 after reattachment surgery (Campo et al., 1999; Pastor et al., 2008). Therapeutic strategies are, therefore, needed that can prevent photoreceptor degeneration and/or stimulate visual recovery.

Mammalian photoreceptors have a highly idiosyncratic energy metabolism. Their inner segments are laden with mitochondria and consume prodigious amounts of oxygen (Cringle et al., 2002) to produce ATP *via* oxidative phosphorylation (Anderson and Saltzman, 1964). Yet, photoreceptors display an aerobic glycolytic profile, with abundant expression of hexokinase II (HK2), pyruvate kinase M2 isozyme (PKM2), lactate dehydrogenase A subunit (LDH-A), and the monocarboxylate transporter MCT-1, as well as the production and release of substantial quantities of lactate (Winkler, 1981; Wang et al., 1997; Gerhart et al., 1999; Lindsay et al., 2014; Casson et al., 2016; Chinchore et al., 2017; Petit et al., 2018). It is reasonable to deduce that a combination of oxidative phosphorylation plus a high glycolytic flux is required for photoreceptor cells to meet their continual energy demands.

A key issue to consider in terms of devising strategies to augment photoreceptor survival and functionality following retinal detachment is understanding what happens to photoreceptor energy metabolism during the detachment process. Surprisingly, to our knowledge, there have been no studies that have investigated the effect of detachment on retinal metabolism in general and photoreceptor energy metabolism in particular. What is known—from experiments performed using felines—is that inner segments display morphological features characteristic of degeneration following retinal detachment, notably disorganisation, swelling, and a marked decrease in mitochondrial cytochrome C oxidase (COX IV) expression (Anderson et al., 1983; Mervin et al., 1999; Lewis et al., 2002). It is logical to infer that loss of COX IV should drastically affect the ability of photoreceptors to survive detachment, yet there may be compensatory changes in other related genes. A further issue that has not been fully resolved is whether lack of oxygen availability or lack of nutrient supply constitutes the major causative factor in photoreceptor injury following retinal detachment. It has been widely presumed that hypoxia plays the major role given the unambiguous findings of Fisher and colleagues (Mervin et al., 1999; Sakai et al., 2001; Lewis et al., 2004), who showed that oxygen supplementation mitigated photoreceptor deconstruction and degeneration following experimental detachment.

The purpose of the present study was to shed light on photoreceptor energy metabolism during experimental detachment in rats. Initially, we investigated whether detachment produced evidence of hypoxia within photoreceptors. Unexpectedly, the findings were negative. Subsequently, we undertook a more detailed appraisal of any alterations in photoreceptor metabolism as a result of detachment, by investigating expression of cellular bioenergetic enzymes. These studies revealed generalised downregulations of both mitochondrial and glycolytic enzymes. Finally, we tested whether oral supplementation with the bioavailable energy substrate pyruvate—the primary fuel input for the citric acid cycle—prevented downregulation of mitochondrial COX IV within inner segments, and mitigated rod and cone deconstruction and degeneration during detachment.

## Materials and methods

### Animals

This study was approved by the Animal Ethics Committee, University of Adelaide (Adelaide, Australia) and conformed with the Australian Code of Practice for the Care and Use of Animals for Scientific Purposes, 2013, and with the ARVO Statement for the use of animals in vision and ophthalmic research. Adult Sprague-Dawley rats (approximately 250 g) were housed in a temperature- and humidity-controlled room with a 12 h light/dark cycle and were provided with food and water *ad libitum*. Ambient lighting was maintained at <50 lux to avoid phototoxicity.

### Experimental model of retinal detachment

The retinal detachment procedure was performed under general anaesthesia (100 mg/kg ketamine plus 10 mg/kg xylazine), following topical instillation of 0.5% tetracaine to provide corneal anaesthesia and 1% tropicamide to facilitate pupil dilation. Initially, an anterior chamber paracentesis was performed *via* the corneal limbus to lower intraocular pressure. Subsequently, a limited superior conjunctival peritomy was fashioned. Bare sclera was exposed and a 32-gauge needle was used to create a sclerotomy located 1.5 mm posterior to the limbus with care taken to avoid lens damage. A bevel down 33-gauge needle was introduced in the sclerotomy and sodium hyaluronate (10 mg/ml, ProVisc, Alcon) was slowly injected into the subretinal space, under direct visualisation of the retina, to detach the neurosensory retina from the underlying retinal pigment epithelium. In all experiments, sufficient hyaluronic acid was injected to ensure that approximately one-half of the neurosensory retina was detached. The other half of the retina remained attached and typically served as an intact sample. Each eye was monitored *via* the operating microscope for a few minutes to ensure that a stable detachment had occurred. Detachments were normally created in one eye of each rat. Any eyes that had surgical complications, specifically choroidal haemorrhage, were excluded from the study.

### Study design

#### Characterisation study

To investigate the effect of retinal detachment upon enzymes involved in energy metabolism, rats subjected to experimental retinal detachment were analysed at various time points: 1 day, 3 days, 1 week, 4 weeks (see Supplementary Table 1 for study design). Some rats received an injection of pimonidazole for localisation of regions of hypoxia (see below).

#### Neuroprotection study

The neuroprotection study comprised two experiments that evaluated whether pyruvate afforded protection against photoreceptor degeneration following retinal detachment. In each experiment, rats were randomly assigned into one of two groups: vehicle (water only) and pyruvate supplementation (500 mg/kg/day in normal drinking water). Rats were commenced on pyruvate supplementation exactly 1 week prior to experimental detachment and continued on pyruvate throughout the experiment. In the first experiment, retinas were analysed 3 days after detachment. This early time point encapsulates the peak of photoreceptor apoptosis and is also when S- and M/L-cone photoreceptor segment loss becomes evident. Retinas were analysed both as wholemounts and transverse sections. In the second experiment, retinas were analysed 4 weeks after detachment. By this later time point, substantial rod photoreceptor loss had occurred. Retinas were analysed as transverse sections (see Supplementary Table 1).

### Spectral domain optical coherence tomography

Non-invasive fundus imaging and spectral domain optical coherence tomography (SD-OCT) were employed in some rats to visualise the spatial extent of retinal detachments, to facilitate dissection of retinas into intact and detached portions for subsequent qPCR analyses, and particularly at the 4-week time point to delineate whether retinas were no longer detached. SD-OCT was performed under general anaesthesia, as above, following topical instillation of 1% tropicamide and 0.5% tetracaine. A custom made hard rodent contact lens (total diameter 5.2 mm, back optic zone radius 2.7 mm, Cantor-Nissel, Brackley, UK), together with lubricating eye gel, was placed on the cornea during the procedure in order to protect against corneal desiccation and to maintain image clarity. Rats were then placed on a custom-designed platform. SD-OCT was performed using the Heidelberg Spectralis (Heidelberg Engineering, Heidelberg, Germany). A wide-field 55° lens was used to capture an overall view of the retina. A series of 97 B-scan SD-OCT images were then taken encompassing the area of retinal detachment. A confocal scanning laser ophthalmoscopy image of the corresponding fundus was also taken.

### Pyruvate bioavailability

The bioavailability of pyruvate in the retinas of vehicle-treated rats and pyruvate-supplemented rats was determined using a commercially available kit (Sigma-Aldrich, Cat# MAK071). Rats (pyruvate = 10, vehicle = 8) were randomly assigned into vehicle (water only) and high dose pyruvate supplementation (500 mg/kg/day in drinking water) groups. They were humanely euthanised on day 14 by transcardial perfusion with physiological saline under terminal anaesthesia and the retinas dissected. Whole retinas were homogenised and the pyruvate level measured.

### Tissue processing

Rats that were to be used for immunohistochemistry on transverse sections were humanely euthanised by transcardial perfusion with physiological saline under terminal anaesthesia. The superior pole of each eye was marked with permanent histological ink for orientation purposes, and the globe was enucleated and immersion-fixed in Davidson’s solution for 24 h before transferring to 70% ethanol until processing. Davidson’s solution, which comprises 2 parts formaldehyde (37%), 3 parts 100% ethanol, 1 part glacial acetic acid, and 3 parts water, is an optimal fixative for retinal detachment studies as it provides excellent tissue morphology while avoiding the processing-induced artefactual retinal detachment that invariably occurs with formalin fixation (Chidlow et al., 2011), thereby permitting precise delineation of the intact and experimentally-induced detached portions of each retina. Eyes were then processed for routine paraffin-embedded sections. Globes were embedded sagittally after careful orientation to ensure that tissue sections were taken through the area of detachment. Correct orientation was achieved by reference to the ink marking and to any accompanying SD-OCT images that had been captured. In all cases, 4 μm sections were cut.

Rats that were to be used for wholemount immunohistochemistry were humanely euthanised by transcardial perfusion with physiological saline under terminal anaesthesia. Eyes were enucleated and fixed in 10% neutral buffered formalin for 24 h before being transferred to phosphate buffered saline (PBS).

### Localisation of hypoxia

To detect cellular hypoxia, 60 mg/kg bodyweight pimonidazole hydrochloride (Hypoxyprobe™-1 kit, Hypoxyprobe Inc, Burlington, MA, USA) diluted in sterile PBS was administered by intraperitoneal injection 3 h prior to euthanasia, as previously described (Gardiner et al., 2005; Mowat et al., 2010; Chidlow et al., 2017). Pimonidazole forms covalent adducts in cells that have an partial pressure of oxygen which is less than 10 mmHg (Arteel et al., 1995). The subsequent staining of tissue sections with an anti-pimonidazole antibody reveals the presence of hypoxic cells (Holcombe et al., 2008; Chidlow et al., 2017). Rats were humanely euthanised by transcardial perfusion with physiological saline, following which they were immersion fixed in Davidson’s solution and processed for paraffin embedding and immunohistochemistry, as described above.

### Histochemical detection of mitochondrial cytochrome C oxidase enzyme activity

Assessment of COX IV was determined using an enzyme histochemistry assay. Eyes from rats (*n* = 3) that had undergone retinal detachment 1 day previously were enucleated and dissected into eyecups. They were then snap frozen in dry ice-cooled isopentane. Unfixed vertical cryosections (9 μm) were taken, air dried and used immediately. Tissue sections were incubated for 40 min at 37°C in a reaction medium containing 5 mg 3,3′-diaminobenzidine, 10 mg of cytochrome C, 60 μg/ml catalase, and 4% sucrose in 10 ml 0.05 M phosphate buffer (pH 7.4). To terminate the reaction, sections were rinsed in distilled water, fixed for 5 min in neutral buffered formalin, and mounted using aqueous mounting medium. Negative control slides, which were processed simultaneously, were performed in the absence of substrate and yielded negligible reaction product.

### Immunohistochemistry

Immunohistochemistry on transverse sections was performed as previously described (Chidlow et al., 2011, 2016). In brief, tissue sections were deparaffinised, endogenous peroxidase activity was blocked and high-temperature antigen retrieval was performed. Subsequently, sections were incubated overnight in primary antibody (Table 1), followed by consecutive incubations with biotinylated secondary antibody and streptavidin-peroxidase conjugate. Colour development was achieved using 3,3′-diaminobenzidine. For double labelling fluorescent immunohistochemistry on transverse sections, visualisation of one antigen was achieved using a 3-step procedure (primary antibody, biotinylated secondary antibody, streptavidin-conjugated AlexaFluor 488 or 594), while the second antigen was labelled by a 2-step procedure (primary antibody, secondary antibody conjugated to AlexaFluor 488 or 594). Sections were prepared as above, then incubated overnight at room temperature in the appropriate combination of primary antibodies. On the following day, sections were incubated with the appropriate biotinylated secondary antibody for the 3-step procedure plus the correct secondary antibody conjugated to AlexaFluor 488 or 594 for the 2-step procedure, followed by streptavidin-conjugated AlexaFluor 488 or 594. Sections were then mounted using anti-fade mounting medium.

**TABLE 1:** Primary antibodies used in the study.

Protein	Source	Clone/Cat. No.	Species	Immunogen	Dilution
AGC1	Cell Signaling Technology	cat# 64169	Rabbit	Synthetic peptide corresponding to residues surrounding Arg309 of human AGC1	1:1,000
mAST	Santa-Cruz	cat# sc-271702	Mouse	aa. 141–211 mapping within an internal region of AATM of human origin	1:1000
COX IV	Molecular Probes	clone 20E8C12	Mouse	Bovine Complex IV (native holoenzyme protein, purified from liver)	1:10,000
CK-MT1A	Proteintech	cat# 15346-1-AP	Rabbit	CKMT1A fusion protein Ag7583	1:5000
FGF-2	Merck-Millipore	clone bFM-2	Mouse	Purified bovine brain basic FGF	1:500
GAPDH	Merck Millipore	clone 6C5	Mouse	Glyceraldehyde-3-phosphate dehydrogenase from rabbit muscle	1:10,000
GFAP	Dako	cat# Z0 334	Rabbit	GFAP isolated from cow spinal cord	1:40,000
Hexokinase II	Cell Signaling Technology	cat# 2867	Rabbit	Synthetic peptide corresponding to the sequence of human hexokinase II	1:500
HIF-1α	Novus	cat# NB100-479	Rabbit	Fusion protein including aa. 530-825 of the mouse HIF-1 alpha protein	1:3000
LDH-A	Santa-Cruz	cat# sc-27230	Goat	Epitope mapping at the N-terminus of LDH-A of human origin	1:1000
LDH-B	Sigma	cat# HPA019007	Rabbit	Recombinant fragment of human LDH-B, aa. 272–334	1:3000
M/L-opsin	Merck-Millipore	cat# AB5405	Rabbit	Recombinant human red/green opsin	1:1500^a^ 1:5000
NSE	Cell Signaling Technology	cat# 8171	Rabbit	Synthetic peptide corresponding to residues near the carboxy terminus of human enolase-2 protein	1:2000
Pimonidazole	Hypoxyprobe Inc	clone 4.3.11.3	Mouse	Pimonidazole adducts	1:500
Phosducin	Santa-Cruz	cat# sc-398752	Mouse	Epitope mapping between aa. 45 and 66 near the N-terminus of human phosducin	1:1000
PKCα	Cell Signaling Technology	cat# 2056	Rabbit	Synthetic peptide corresponding to human PKCα	1:300^a^
PKM2	Cell Signaling Technology	cat# 4053	Rabbit	Synthetic peptide corresponding to the sequence of human PKM2	1:2500
S-opsin	Santa-Cruz	cat# sc-14363	Goat	Peptide mapping at the N-terminus of the opsin protein encoded by OPN1SW of human origin	1:1500^a^
Rhodopsin	Santa-Cruz	clone RET-P1	Mouse	Membrane preparation from adult rat retina	1:1000
SOD-2	Antibody Technology Australia Pty Ltd	cat# SOD2R	Rabbit	Human/rat/mouse SOD2 aa. 25–43	1:10,000
SUCLA2	Proteintech	cat# 12627-1-AP	Rabbit	SUCLA2 fusion protein Ag3319	1:1000

^a^Dilution used for 2-step fluorescent immunostaining procedure.

Double labelling wholemount immunohistochemistry was performed as previously described (Chidlow et al., 2022). In brief, globes were dissected into posterior eye-cups, and retinas were prepared as flattened wholemounts by making four radial cuts. Retinas were then incubated sequentially in PBS containing 1% Triton X-100 (PBS-T) for 1 h, PBS-T containing 3% normal horse serum (NHS-T) for 1 h to block non-specific antibody binding, and a combination of anti-S-opsin and anti-M/L-opsin antibodies diluted in NHS-T for 2 days at 4°C (Table 1). On the third day, retinas were washed in PBS-T and incubated overnight at 4°C with a combination of AlexaFluor-488 and -594 conjugated secondary antibodies. Finally, retinas were washed in PBS prior to mounting with the photoreceptor side facing up using anti-fade mounting medium.

Confirmation of the specificity of antibody labelling was judged by the morphology and distribution of the labelled cells, by the presence of an analogous immunolabeling pattern when a primary antibody directed against a different epitope was used, by the absence of signal when the primary antibody was replaced by isotype/serum controls, and by comparison with the expected staining pattern based on our own, and other, previously published results.

### TUNEL assay

Tissue sections were deparaffinised, rehydrated and rinsed in PBS. Next, sections were treated with proteinase K (10 μg/ml) for 7 min, followed by three rinses of distilled water for 2 min. Sections were then equilibrated in TdT buffer (30 mM Tris-HCl, pH 7.2 containing 140 mM sodium cacodylate, 1 mM cobalt chloride), prior to incubation for 1 h at 37°C in the same buffer containing TdT (0.15 U/μl) and biotin-16-dUTP (10 μM). The reaction was terminated by two washes of 15 min in saline sodium citrate solution. Following a rinse in PBS, non-specific binding sites were blocked using 2% bovine serum albumin, prior to incubation of sections with streptavidin Alexafluor 594 conjugate for 30 min to visualise TUNEL positive cells. After rinsing in PBS, sections were counterstained with DAPI, rinsed in PBS and then coverslipped using anti-fade mounting medium.

### Quantification of histopathology

All analyses were conducted in a blinded fashion. Fluorescent immunohistochemistry was examined under a fluorescence microscope (BX-61; Olympus, Mount Waverley, VIC, Australia) equipped with a scientific grade, cooled CCD camera. Colorimetric immunohistochemistry was examined under a light microscope (BX51, Olympus) equipped with a DP20 digital camera.

#### Wholemounts

For quantification of the temporal patterns of S-cone and M/L-cone segment loss, photomicrographs (measuring 1130 × 845 μm) of detached and intact portions of each wholemount retina were captured. Quantification of cone survival was performed using Image-J software (NIH, Bethesda, MD, USA). Initially, however, images were processed in Photoshop CS3 (Adobe). Images were corrected for uneven lighting using a flatten filter and where necessary linear gradient tool, then sharpened, levels enhanced, and converted to 8-bit mode. Images were analysed both for number of cone segments and for total area of cone segments with the Image-J “analyse particles” function using a minimum size of 5 square pixels.

#### Transverse sections

For all quantifications from colorimetric and fluorescent immunolabeling as well as the TUNEL assay, 20x objective lens magnification, non-overlapping photomicrographs were captured from intact and detached regions of each retina. Owing to the fact that detached areas of retina adjacent to intact retina appeared relatively more normal than detached areas situated further from the intact retina, all photomicrographs were taken at a minimum distance of 0.5 mm from the intact retina. The number of photomicrographs captured of each detached retina varied, but was typically 2–3. For TUNEL, the number of positively labelled cells per mm^2^ of the outer nuclear layer (ONL) was quantified. For phosducin, COX IV and HK2 the total integrated density of immunolabeling of the ONL plus photoreceptor segments (phosducin) or photoreceptor segments alone (COX IV, HK2) was quantified per photomicrograph using the Image-J “analyse particles” function. For GFAP and M/L-opsin, the total area of immunolabeling of cone segments (M/L-opsin) or throughout the retina (GFAP) was quantified per photomicrograph using the Image-J “analyse particles” function. Measurement of the thickness of the ONL was achieved using the Image-J “Measure” tool. To ensure consistency between samples, values were normalised to the thickness of the inner retina to account for any errors caused by different angles of sectioning. For LDH-A, GAPDH, PKM2 and FGF-2, the mean intensity of immunolabeling within the ONL per photomicrograph was analysed by densitometry using the program, Adobe PhotoShop CS2.

#### Statistical analyses

For the characterisation study, the null hypothesis tested was that the levels of immunohistochemical markers at each time point following experimental detachment would be unchanged from intact retinas. Statistical analysis was carried out by one-way ANOVA followed by *post-hoc* Dunnett’s multiple comparisons test. For the neuroprotection study, the null hypothesis tested was that following retinal detachment the number of TUNEL-positive cells, the thickness of the ONL, and the levels of expression of COX IV, M/L-opsin, S-opsin, GFAP and phosducin in pyruvate- vs. vehicle-treated would be the same. In each case, statistical analysis was carried out by Student’s unpaired *t*-test followed by modified Bonferroni correction. For the pyruvate bioavailability experiment, the null hypothesis tested was that the level of retinal pyruvate would be unchanged in oral pyruvate-supplemented rats as compared to rats administered water only. Statistical analysis was carried out by Student’s unpaired *t*-test.

### qPCR

Quantitative RT-PCR (qPCR) studies were carried out as described previously (Chidlow et al., 2014). In brief, entire retinas and optic nerves were dissected, total RNA was isolated and first strand cDNA was synthesised from DNase-treated RNA. Real-time PCR reactions were carried out in 96-well optical reaction plates using the cDNA equivalent of 20 ng total RNA for each sample in a total volume of 20 μl containing 1 × SYBR Green PCR master mix (Bio-Rad, Gladesville, NSW, Australia) and forward and reverse primers. The thermal cycling conditions were 95°C for 3 min and 40 cycles of amplification comprising 95°C for 12 s, annealing temperature (Supplementary Table 2) for 30 s and 72°C for 30 s. After the final cycle of the PCR, primer specificity was checked by the dissociation (melting) curve method. PCR assays were performed using the CFX cycler (Bio-Rad) and all samples were run in duplicate. Threshold cycles were calculated using CFX Manager Software (Bio-Rad). All values were normalised using a pool of two endogenous reference genes, cyclophilin and hypoxanthine phosphoribosyltransferase 1 (HPRT1), using The *BestKeeper* software tool (Pfaffl et al., 2004) and expressed as mean ± SEM. Primer pairs are shown in Supplementary Table 2. The results showed that all mRNAs were amplified with high efficiency and linearity during real-time PCR. Mean amplification efficiencies, as determined by plotting cycle threshold as a function of initial cDNA quantity, ranged from 1.9—2.0. Results obtained were, therefore, quantified using the comparative threshold cycle (C_T_) method (ΔΔC_T_) for relative quantitation of gene expression, with a minor correction for amplification efficiency (Pfaffl, 2001). The null hypothesis tested was that the mRNA levels of all genes of interest (when normalised to housekeeping genes) in detached retinal samples would be the same as in intact retinal samples. Statistical analysis was carried out by Student’s unpaired *t*-test followed by modified Bonferroni correction.

### Mixed retinal cell culture

Rat retinal cell cultures comprising glia, photoreceptors, and neurons, were prepared from the pups *via* a trypsin- and mechanical-digest procedure as previously described (Wood et al., 2012). In brief, after tissue dissociation, cells were dispensed onto 13-mm diameter glass coverslips coated with poly-L-lysine (10 μg/mL, 15 min) in 24-well culture plates. Mean cell density at seeding was approximately 0.5 × 10^6^ cells/ml. Subsequently, cultures were grown at 37°C in a humidified incubator with 5% CO_2_ in growth medium. After 6 days, medium was changed and the cultures were incubated for 24 h with either standard DMEM medium, or with DMEM medium lacking glucose, glutamine, pyruvate, and serum (nutrient deprivation), or with nutrient deprivation DMEM medium but with added pyruvate (50 μM to 5 mM). Cultures were then fixed in 10% neutral buffered formalin for immunocytochemical analysis. Statistical analyses were carried out by ANOVA followed by *post-hoc* Dunnett’s multiple comparisons test.

### Branch retinal vein occlusion

Branch retinal vein occlusion (BRVO) was performed under general anaesthesia, as detailed above, following topical instillation of 0.5% tetracaine and 1% tropicamide. All rats then underwent laser photocoagulation across three evenly spaced retinal veins in both eyes. After being anaesthetised, they received a 0.25 ml intravenous injection of Rose Bengal (4,5,6,7-tetrachloro-2′,4′,5′,7′-tetraiodo-fluorescein). They were then placed within 1 min of injection on a custom mount attached to a slit lamp with a 532-nanometre laser provided by Ellex R&D Pty. Ltd. (Adelaide, SA, Australia). A small fundus laser lens for mice (Ocular Instruments, Inc., Bellevue, WA, USA) was used to visualise the fundus and blood vessels during the laser application. The laser was used to occlude the retinal veins approximately two to three optic disc diameters away from the centre of the retina using settings of 100 mW power, 50 μm spot size and 800 ms exposure. Each retinal vein received sufficient laser spots to be fully occluded.

## Results

### Retinal detachment does not cause measureable hypoxia within photoreceptors

**FIGURE 1:**
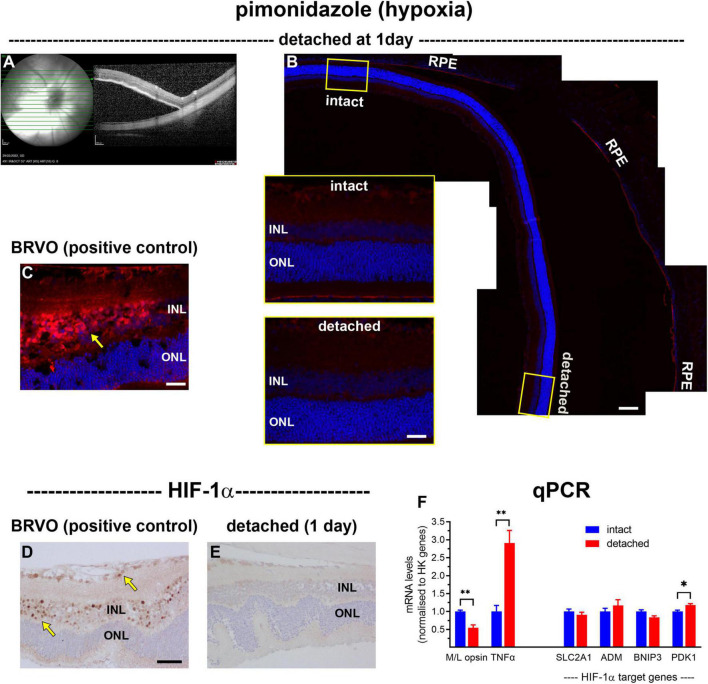
Lack of hypoxia, HIF-1α accumulation, and HIF-1α target gene upregulation following retinal detachment. **(A)** Visualisation of representative retinal detachment at 1 day by fundus imaging and SD-OCT. **(B,C)** Representative images of pimonidazole staining (red, arrow) in retinas subjected to experimental detachment or branch retinal vein occlusion (BRVO). **(D,E)** Representative images of HIF-1α immunolabelling in retinas subjected to experimental detachment or BRVO (arrows). Scale bars: **(B)** (overview) = 120 μm; **(B)** inset, **(C)** = 30 μm; **(D,E)** = 60 μm. INL, inner nuclear layer; ONL, outer nuclear layer; RPE, retinal pigment epithelium. **(F)** Quantification of various mRNA levels at 1 day after detachment. Values (mean ± SEM) are normalised to a pool of two housekeeping genes and expressed as % of the intact group. *P < 0.05, ^**^P < 0.01 by Student’s unpaired *t*-test followed by modified Bonferroni correction.

The first objective of the study was to investigate whether experimental detachment causes retinal hypoxia, using the exogenous hypoxia marker pimonidazole, the endogenous marker hypoxia-inducible factor-1α (HIF-1α), as well as by qPCR analysis of HIF target genes. Pimonidazole forms stable covalent adducts with proteins in cells with a pO_2_ of less than 10 mmHg (Arteel et al., 1995), which can then be localised by immunohistochemical labelling in fixed tissue sections. At 1 day after surgery, all detachments were bullous, as identified by fundus imaging and SD-OCT (Figure 1A). As expected, no pimonidazole labelling was evident in intact retinas (Figure 1B). Surprisingly, no unambiguously-positive staining for pimonidazole was identified within any of the detached retinas (Figure 1B). To validate the pimonidazole methodology to detect retinal hypoxia, we used tissue blocks from rats subjected to branch retinal vein occlusion (BRVO). In contrast to retinal detachment, at 1 day after BRVO, the inner retina layers stained strongly for pimonidazole (Figure 1C). Similar results were found using HIF-1α. In BRVO retinas, nuclear labelling for HIF-1α was evident within cells of the inner retina (Figure 1D), whereas no HIF-1α expression was observed in detached retinas (Figure 1E). Finally, we investigated whether there was upregulation of recognised HIF target genes in retinas detached for 1 day (Figure 1F). This approach was facilitated by the use of fundus imaging and SD-OCT. With reference to these images, each posterior eye-cup was carefully hemisected into detached and intact portions, and the retinal samples taken for mRNA extraction. The data showed that expression of the mRNA encoding the cone outer segment protein M/L-opsin in detached samples was downregulated to 55 ± 8% of intact retina, while the mRNA encoding the neuroinflammatory marker TNFα was upregulated to 291 ± 35% of intact retina. There were no significant changes in the levels of BNIP3, ADM, or SLC2A1 mRNAs in detached samples compared to intact samples, with a small (18 ± 4%) but significant upregulation in PDK1. These overall results show that, in the rat, experimental detachment does not lead to a reduction in pO_2_ within the outer retina that is of sufficient magnitude to produce covalent adducts of pimonidazole or nuclear expression of HIF-1α, or consistent upregulation of HIF target genes.

### Retinal detachment has a profound effect upon mitochondria

**FIGURE 2:**
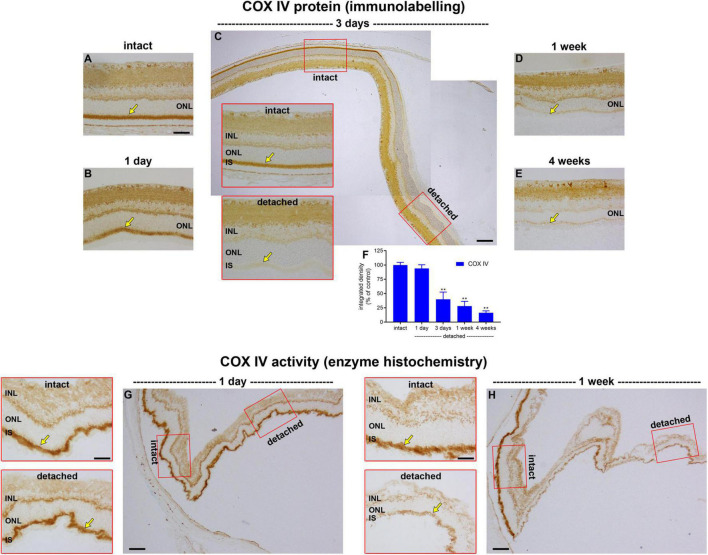
Effect of retinal detachment on expression and activity of cytochrome C oxidase (COX IV), as evaluated by immunohistochemistry and enzyme histochemistry. **(A–E)** Representative images of COX IV immunolabelling in intact retina, and at 1 day, 3 days, 1 week, and 4 weeks after detachment. Arrows demarcate inner segments (IS). **(F)** Quantification of COX IV immunoreactivity associated with IS at 1 day, 3 days, 1 week, and 4 weeks after retinal detachment. Values, shown as % of the intact group, represent mean ± SEM. **P < 0.01 by one-way ANOVA followed by *post-hoc* Dunnett’s multiple comparisons test (intact vs. detached). **(G,H)** Representative images of COX IV activity as evaluated by enzyme histochemistry in intact retina and at 1 day and 1 week after detachment. Scale bars: **(A–C)** (insets), **(D,E,G)** (insets), and **(H)** (insets) = 60 μm; **(C,G,H)** (overviews) = 150 μm. INL, inner nuclear layer; ONL, outer nuclear layer.

Given that retinal detachment does not cause photoreceptor hypoxia, as evidenced by the lack of pimonidazole staining or HIF1α accumulation, we investigated whether there is a marked loss of mitochondrial COX IV within inner segments, as occurs in the detached cat retina (Mervin et al., 1999). To achieve this goal, we performed immunohistochemistry on transverse sections of the retina at successive time points after detachment, and then quantified the amount of COX IV. In intact retinas, immunoreactivity for COX IV was characterised by punctate labelling of both plexiform layers, together with intense labelling of rod and cone inner segments (Figure 2A). As expected, there was no discernible change in the pattern of COX IV immunolabeling within the inner retinal layers. Immunolabeling of photoreceptor inner segments was largely unaltered at 1 day after detachment (*P* = 0.92; Figures 2B,F), but had decreased substantially to 40.3 ± 12.6% of intact retina by 3 days (*P* < 0.01; Figures 2C,F), to 27.8 ± 8.4% by 1 week of detachment (*P* < 0.01; Figures 2D,F), and to 16.3 ± 3.3% of intact retina by 4 weeks (*P* < 0.01; Figures 2E,F).

The results show that there is a time window of at least 24 h from detachment until COX IV expression begins to be lost. It is important to know whether the enzyme remains fully functional during this time period or whether it has already undergone structural damage that compromises its activity. To address this question, we examined COX IV activity in retinas detached for 1 day, using enzyme histochemistry in unfixed tissue sections. It is vital to stress that such an assay does not inform as to how much COX IV activity might actually be occurring in the detached retina—for the simple reason that the assay itself provides the necessary substrate and oxygen for the enzyme to function, which may very well not be the case *in vivo*—it simply sheds light on whether, given sufficient substrate and oxygen, the enzyme performs efficiently. The data showed that intensity of enzyme reaction in the inner segments appeared similar in intact and detached portions of retina (Figure 2G), indicating that mitochondrial COX IV activity retains its functionality for at least 1 day of detachment. Unlike 1 day, analysis of COX IV activity in retinas detached for 1 week showed a marked reduction in intensity (Figure 2H), results in line with the immunohistochemical findings.

**FIGURE 3:**
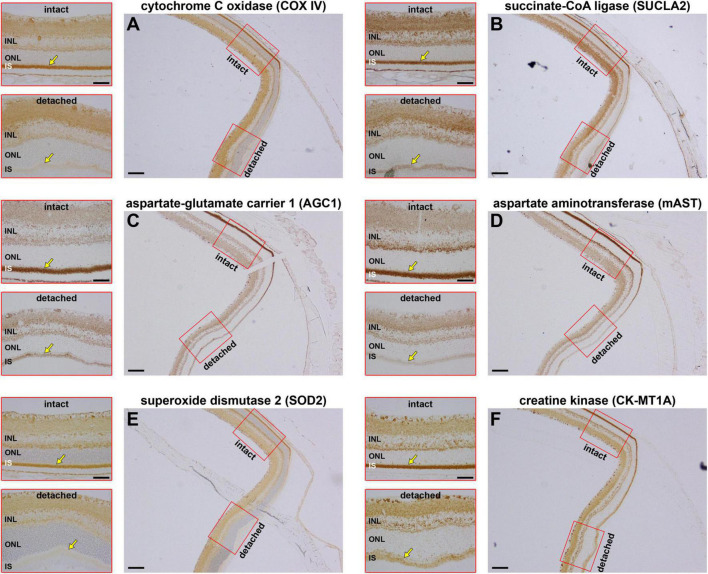
Effect of retinal detachment on expression of six mitochondrial enzymes within the same 3 days detached retina. **(A)** Cytochrome C oxidase (COX IV). **(B)** Succinate-CoA ligase (SUCLA2). **(C)** Aspartate-glutamate carrier 1 (AGC1). **(D)** Mitochondrial aspartate aminotransferase (mAST). **(E)** Mitochondrial superoxide dismutase (SOD2). **(F)** Mitochondrial creatine kinase (CK-MT1A). Scale bar: overviews = 150 μm; insets = 60 μm. INL, inner nuclear layer; SI, inner segments; ONL, outer nuclear layer.

COX IV is the final enzyme in the respiratory electron transfer chain and is necessarily dependent upon sufficient oxygen to function. If oxygen is scarce, or completely absent, then mitochondria can still generate ATP by substrate-level phosphorylation, a mechanism independent of the proton motive force. Succinate-CoA ligase (SUCL), a heterodimeric enzyme of the citric acid cycle enzyme, catalyses substrate-level phosphorylation and can maintain matrix ATP levels under energy-deficient conditions, such as hypoxia (Weinberg et al., 2000; Chinopoulos and Seyfried, 2018). We examined the effect of retinal detachment upon expression of SUCLA2, the ATP-forming β-subunit of SUCL. The results showed that expression of SUCLA2 was lost in a very similar fashion to that of COX IV, with minimal change at 1 day (data not shown), but increasing loss of SUCLA2 thereafter (Figure 3).

The COX IV and SUCLA2 findings suggest that there is a generalised loss of inner segment mitochondrial proteins after retinal detachment. To substantiate this hypothesis, we assessed the effect of detachment upon expression of four additional mitochondrial proteins, namely the antioxidant enzyme superoxide dismutase 2 (SOD2), an abundant protein that is localised to the mitochondrial matrix; mitochondrial aspartate-glutamate carrier 1 (AGC1) and mitochondrial aspartate aminotransferase (mAST), both of which are part of the malate-aspartate shuttle; and mitochondrial creatine kinase (CK-MT1A), an enzyme known to be abundant in photoreceptor inner segments. Expression patterns for COX IV (Figure 3A) and SUCLA2 (Figure 3B), plus the other four mitochondrial proteins (Figures 3C–F), are shown in tissue sections from the same 3 days detached retina. The results show downregulated expression/loss of immunolabeling for all six proteins. The extent of loss appears considerable for each protein. The data emphasise that there appears to be a substantial loss of all mitochondrial proteins following detachment, indicating that the functional status of inner segment mitochondria is likely to be majorly impaired.

### Retinal detachment does not cause a compensatory upregulation in glycolysis

The preceding data set has shown that photoreceptor mitochondrial bioenergetics are substantially compromised by ongoing retinal detachment. The next question we sought to address, therefore, was whether there is a compensatory upregulation in glycolytic enzymes, i.e., whether the detachment-induced loss of oxidative phosphorylation causes a Pasteur-like effect. Such analysis is, of course, complicated by the fact that photoreceptors already display a Warburg effect—producing substantial quantities of lactate and expressing high levels of glycolytic genes associated with both the Pasteur and Warburg effects under normal physiological conditions (Winkler, 1981; Wang et al., 1997; Gerhart et al., 1999; Lindsay et al., 2014; Casson et al., 2016; Chinchore et al., 2017; Petit et al., 2018).

**FIGURE 4:**
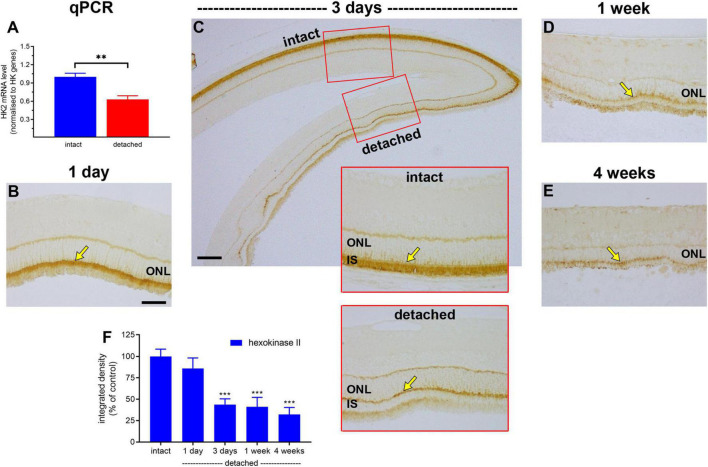
Effect of retinal detachment on expression of hexokinase II, as evaluated by qPCR and immunohistochemistry. **(A)** Quantification of HK2 mRNA level at 1 day after detachment. Values (mean ± SEM) are normalised to a pool of two housekeeping genes and expressed as % of the intact group. **P < 0.01 by Student’s unpaired *t*-test. **(B–E)** Representative images of hexokinase II immunolabelling in intact retina, and at 1 day, 3 days, 1 week, and 4 weeks after detachment. Arrows highlight photoreceptor inner segments. **(F)** Quantification of hexokinase II immunoreactivity associated with inner segments (IS) at 1 day, 3 days, 1 week, and 4 weeks after retinal detachment. Values, shown as % of the intact group, represent mean ± SEM. ***P < 0.001 by Student’s unpaired *t*-test followed by modified Bonferroni correction (intact vs. detached). Scale bars: **(A–C)** (insets), **(D)** = 60 μm; **(C)** (overview) = 150 μm. ONL, outer nuclear layer.

Hexokinase catalyses the first step of glycolysis. In healthy photoreceptors, the two major isoforms, HK1 and HK2, are mainly bound to mitochondria within inner segments (Rueda et al., 2016), which facilitates coupling between glycolysis and oxidative phosphorylation. HK2 constitutes the primary regulated isoform of hexokinase and its expression, as well as cellular distribution, undergo dynamic change in conditions of metabolic stress (Mathupala et al., 2001; John et al., 2011). We anticipated a marked loss of HK2 from photoreceptor inner segments following detachment. The results showed this to be the case. In intact retinas, HK2 expression was expressed by photoreceptor inner segments and, at lower levels, their axon terminals in the outer plexiform layer (Figure 4). At 1 day after detachment, qPCR analysis showed expression of HK2 mRNA in detached samples had decreased to 63% of the intact retina (Figure 4A), although there was no significant reduction in HK2 immunolabeling (*P* = 0.56; Figures 4B,F). By 3 days after detachment, HK2 immunolabeling had decreased to 43.7 ± 6.7% of intact retina (*P* < 0.01; Figures 4C,F), by 1 week to 41.2 ± 10.9% of detachment (*P* < 0.01; Figures 4D,F) and by 4 weeks to 32.2 ± 8.2% of intact retina (*P* < 0.01; Figures 4E,F). Of note, there was no prominent translocation of HK2 to the cytosolic compartment of photoreceptors located within the ONL; however, a residual band of HK2 labelling was typically evident at the junction of the ONL and inner segments.

**FIGURE 5:**
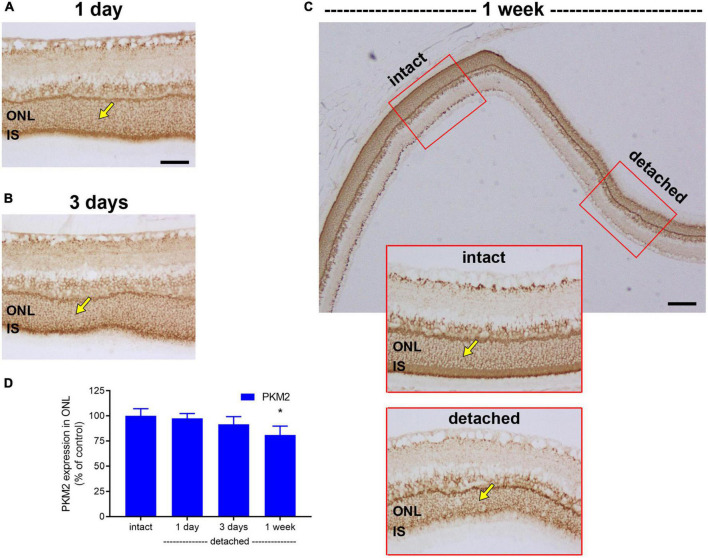
Effect of retinal detachment on expression of pyruvate kinase M2 isozyme (PKM2), as evaluated by immunohistochemistry. **(A–C)** Representative images of PKM2 immunolabelling in intact retina, and at 1 day, 3 days, and 1 week after detachment. Arrows demarcate photoreceptor cell bodies. **(D)** Quantification of PKM2 immunoreactivity within the outer nuclear layer (ONL) at 1 day, 3 days, 1 week, and 4 weeks after retinal detachment. Values, shown as % of the intact group, represent mean ± SEM. *P < 0.05 by Student’s unpaired *t*-test followed by modified Bonferroni correction (intact vs. detached). Scale bars: **(A–C)** (insets) = 60 μm; **(C)** (overview) = 150 μm. IS, inner segments.

Next, we evaluated expression of PKM2. PKM2 is a cytosolic enzyme that catalyses the last irreversible step in glycolysis and is a direct HIF-1 target gene (Luo et al., 2011). In intact retina, both photoreceptor cell somas in the ONL and their inner segments robustly expressed PKM2 (Figure 5). Following detachment, PKM2-labelled inner segments underwent partial degeneration. Intensity of PKM2 immunolabeling within the ONL did not increase after detachment, but the level was largely maintained, decreasing gradually to 97.3 ± 4.9% of intact retina at 1 day (*P* = 0.98; Figures 5A,D), 91.4 ± 7.7% of intact retina at 3 days (*P* = 0.55; Figures 5B,D) and 80.9 ± 8.8% of intact retina at 1 week (*P* = 0.07; Figures 5C,D).

**FIGURE 6:**
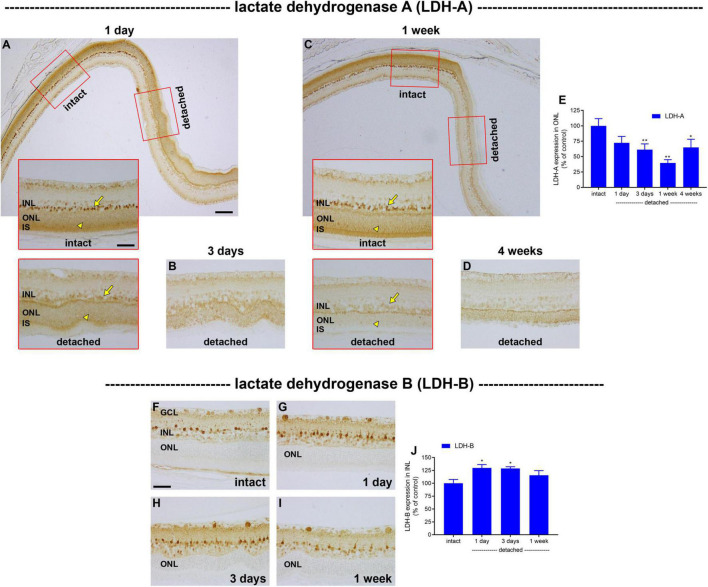
Effect of retinal detachment on expression of lactate dehydrogenase (LDH)-A and -B subunits, as evaluated by immunohistochemistry. **(A–D)** Representative images of LDH-A immunolabelling in intact retina, and at 1 day, 3 days, 1 week, and 4 weeks after detachment. Arrows highlight bipolar cells; arrowheads demarcate photoreceptor cell bodies. **(E)** Quantification of LDH-A immunoreactivity within the outer nuclear layer (ONL) at 1 day, 3 days, 1 week, and 4 weeks after retinal detachment. Values, shown as % of the intact group, represent mean ± SEM. *P < 0.05, **P < 0.01 by one-way ANOVA followed by *post-hoc* Dunnett’s multiple comparisons test (intact vs. detached). Scale bars: **(A)** (insets), **(B,C)** (insets), **(D)** = 60 μm; **(A,C)** (overview) = 150 μm. **(F–I)** Representative images of LDH-B immunolabelling in intact retina, and at 1 day, 3 days, and 1 week after detachment. Scale bar: 60 μm. **(J)** Quantification of LDH-B immunoreactivity within the inner nuclear layer (INL) at 1 day, 3 days, 1 week, and 4 weeks after retinal detachment. Values, shown as % of the intact group, represent mean ± SEM. Values, shown as % of the intact group, represent mean ± SEM. *P < 0.05 by one-way ANOVA followed by *post-hoc* Dunnett’s multiple comparisons test (intact vs. detached).

Lactate dehydrogenase (LDH), which catalyses the interconversion of pyruvate to lactate, is an isoenzyme composed of LDH-A and/or LDH-B subunits. LDH-A subunits favour the formation of lactate, whereas LDH-B favours the reverse reaction. Increased lactate production *via* LDH-A is one of the defining features of the metabolic switch from oxidative phosphorylation to glycolysis (Semenza et al., 1996). In intact retina, LDH-A localised to a population of cells in the outer part of the inner nuclear layer and was also strongly expressed by photoreceptor cell bodies in the ONL and their inner segments (Figures 6A,C). Double labelling immunofluorescence experiments showed that there was an almost perfect colocalisation of LDH-A immunoreactivity in the inner nuclear layer with the bipolar cell marker PKCα (Supplementary Figure 1). Following detachment, there was a prolonged reduction in expression of LDH-A by photoreceptor somas and inner segments that was more marked than that of PKM2. The intensity of LDH-A immunolabeling within the ONL decreased to 72.3 ± 10.4% of intact retina at 1 day (*P* = 0.09; Figures 6A,E), 61.2 ± 9.5% of intact retina at 3 days (*P* < 0.01; Figures 6B,E) and 39.6 ± 5.4% of intact retina at 1 week (*P* < 0.01; Figures 6C,E), before recovering to 64.9 ± 13.3 by 4 weeks (*P* = 0.02; Figures 6D,E). Curiously, the pattern of LDH-A expression in bipolar cells mirrored that of photoreceptors, despite the fact that bipolar cells retain access to their vascular supply, with a striking and prolonged decrease after detachment (Figures 6A–D). For comparative purposes, we also evaluated expression of LDH-B after detachment. In intact retina, LDH-B was expressed by cell bodies and their processes within the inner retina, but was not observed in photoreceptors (Figure 6F). Detachment caused a modest, but significant, elevation of LDH-B expression within the inner retina at 1 day (Figures 6G,J) and 3 days (Figures 6H,J) but not 1 week (Figure 6I) after detachment, but this enzyme remained undetectable in photoreceptors. The lack of LDH-B reduces the possibility of photoreceptors using lactate as a fuel, although since both LDH-A and LDH-B are not strictly unidirectional enzymes, the presence of LDH-A in photoreceptors would theoretically allow the generation of pyruvate if the local lactate concentration is high.

**FIGURE 7:**
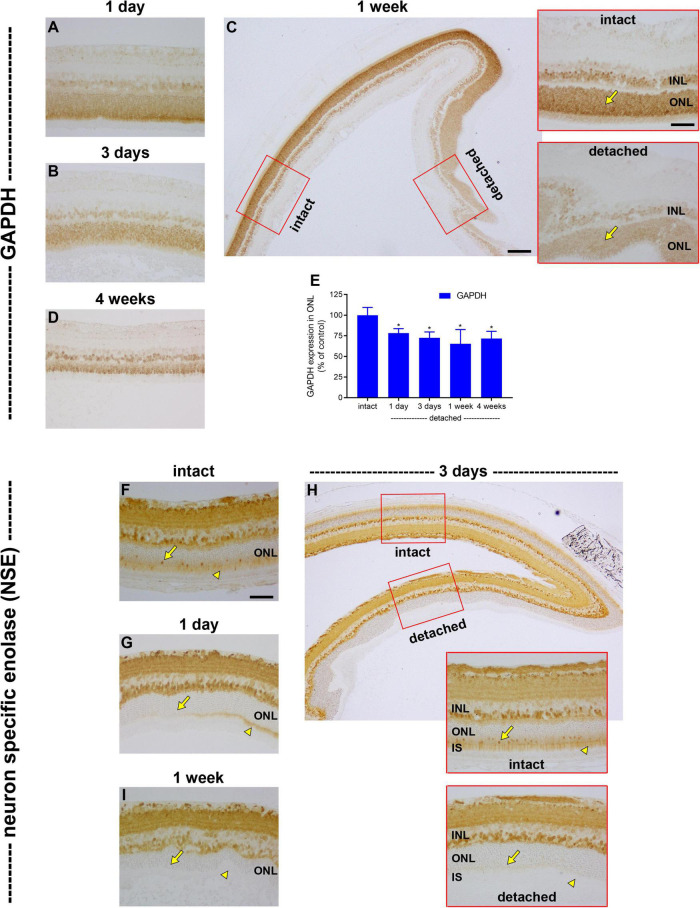
Effect of retinal detachment on expression of GAPDH and neuron specific enolase (NSE), as evaluated by immunohistochemistry. **(A–D)** Representative images of GAPDH immunolabelling in intact retina, and at 1 day, 3 days, 1 week, and 4 weeks after detachment. Scale bars: **(A,B)** (insets) = 60 μm; **(B)** (overview) = 150 μm. **(E)** Quantification of GAPDH immunoreactivity within the ONL at 1 day, 3 days, 1 week, and 4 weeks after retinal detachment. Values, shown as % of the intact group, represent mean ± SEM. *P < 0.05 by Student’s unpaired *t*-test followed by modified Bonferroni correction (intact vs. detached). **(F–I)** Representative images of NSE immunolabelling in intact retina, and at 1 day, 3 days, and 1 week after detachment. In each case, arrowheads highlight photoreceptor inner segments; arrows demarcate photoreceptor cell bodies. Scale bars: **(D–F)** (insets), **(G)** = 60 μm; **(F)** (overview) = 150 μm.

The prolonged downregulation of LDH-A in photoreceptors after detachment indicates that there is no compensatory increase in glycolysis leading to lactate production, despite the disruption to oxidative phosphorylation. To substantiate this deduction, we analysed expression of GAPDH, a key cytosolic enzyme that exists as a single isoform. In intact retina, GAPDH was principally associated with bipolar cells and photoreceptor cell bodies (Figure 7). Following detachment, there was a prolonged reduction in expression of GAPDH by photoreceptor somas and inner segments that was greater than that of PKM2, but less marked than LDH-A. The intensity of GAPDH immunolabeling within the ONL decreased to 78.2 ± 5.5% of intact retina at 1 day (*P* < 0.05; Figures 7A,E), 72.5 ± 7.1% of intact retina at 3 days (*P* < 0.05; Figures 7B,E) and 65.2 ± 17.3% of intact retina at 1 week (*P* < 0.05; Figures 7C,E), before recovering to 71.6 ± 8.8 by 4 weeks (*P* < 0.05; Figures 7D,E). Finally, we investigated expression of the glycolytic enzyme neuron specific enolase (NSE). NSE is a useful marker to evaluate because it is detectable in cone but not rod somas (Rich et al., 1997). In intact retina, NSE was present in inner retinal neurons, rod and cone inner segments and cone somas (Figure 7F). Following detachment, there was an early, striking, and prolonged loss of NSE from inner segments as well as cone somas (Figures 7G–I). NSE-labelled cones were essentially not detectable from as early as 1 day after detachment. NSE labelling of the inner retina was unaffected by detachment (Figures 7G–I).

### Photoreceptors remain capable of *de novo* protein synthesis

**FIGURE 8:**
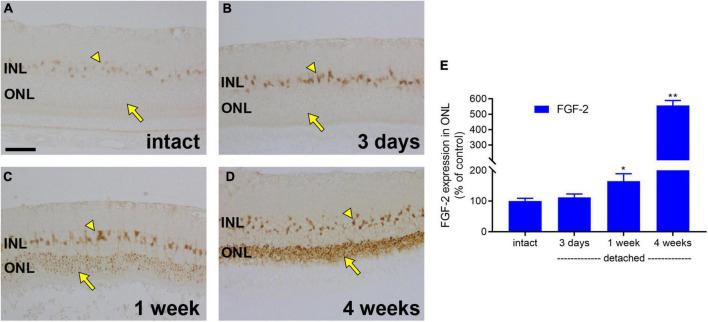
Retinal detachment causes induction of FGF-2 expression in detached photoreceptors. **(A–D)** Representative images of FGF-2 immunolabelling in intact retina, and at 3 days, 1 week, and 4 weeks after detachment. Arrowhead highlights Müller cell somas; arrow demarcates photoreceptor cell bodies. Scale bar: 60 μm. **(E)** Quantification of FGF-2 immunoreactivity within the outer nuclear layer (ONL) at 3 days, 1 week, and 4 weeks after retinal detachment. Values, shown as % of the intact group, represent mean ± SEM. *P < 0.05, **P < 0.01 by Student’s unpaired *t*-test followed by modified Bonferroni correction (intact vs. detached). INL, inner nuclear layer.

We have provided evidence for a profound loss of mitochondrial bioenergetic capacity, as well as for a downregulation of glycolytic enzymes, after retinal detachment. We were also interested in whether photoreceptors retained sufficient energetic reserves to upregulate proteins that may aid their survival under the challenging environment of detachment. To test this hypothesis, we evaluated expression of the endogenous trophic factor FGF-2. In intact retina, weak FGF-2 labelling was observed in Müller cell somas (Figure 8A). At 1 day (data not shown) and 3 days (Figures 8B,E), after detachment, there was a discernible upregulation in Müller cells, but no evidence of expression by photoreceptors. By 1 week, there was a measureable expression of FGF-2 by photoreceptor somas (164.2 ± 24.8% of intact retina; Figures 8C,E), which had increased to 556.2 ± 33.5% of intact retina by 4 weeks (Figures 8D,E).

### Supplementation with pyruvate fails to protect against photoreceptor degeneration following retinal detachment

**FIGURE 9:**
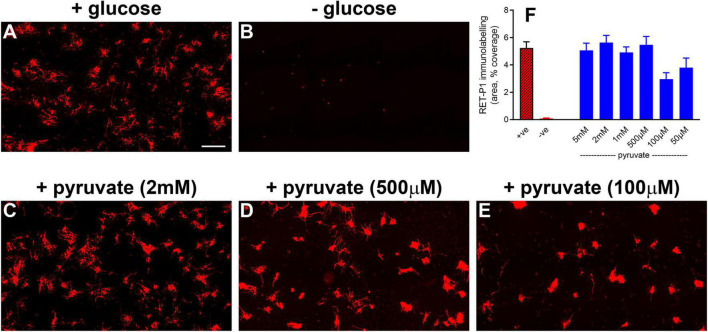
Pyruvate protects against hypoglycemia-induced death of photoreceptors in mixed retinal cultures. **(A–E)** Representative images of RET-P1 (rhodopsin) immunolabelling in mixed retinal cultures subjected to normal medium **(A)**, nutrient deprivation **(B)**, or nutrient deprivation plus pyruvate **(C–E)**. Scale bar: 200 μm. **(F)** Quantification of RET-P1 immunolabelling. Values, shown as % of the +glucose group, represent mean ± SEM.

The final and major goal of this study was to test whether oral supplementation with pyruvate was neuroprotective to rod and cone photoreceptors during retinal detachment. The rationale for using pyruvate was as follows: our results herein have shown that detachment does not cause hypoxia in the outer retina, but does lead to downregulated expression of bioenergetic enzymes by photoreceptors. These results can be interpreted as suggesting that ongoing substrate insufficiency may be critical in terms of pathogenesis. Accordingly, boosting metabolic inputs may preserve the bioenergetic capacity of photoreceptors and prevent their deconstruction and degeneration. Initially, we tested whether pyruvate can rescue photoreceptors from nutrient deprivation in mixed retinal cultures. The results showed an almost complete preservation of immunolabeled photoreceptors when supplemented for 24 h with pyruvate ranging from 500 μM to 5 mM in the absence of alternative substrates for energy production (Figures 9A–D,F). Lower concentrations of pyruvate, 100 μM and 50 μM, also preserved photoreceptor survival as compared to nutrient-deprived cultures (Figures 9E,F; *P* < 0.01 pyruvate vs. nutrient-deprived), but did not provide complete protection. Moreover, scrutiny of the cultures treated with 50 μM and 100 μM pyruvate showed that surviving neurons displayed fewer fine processes when compared to higher doses of pyruvate (Figure 9E). Next, we measured the retinal bioavailability of pyruvate after oral supplementation for 2 weeks in healthy rats. The results showed a statistically significant (*P* < 0.01) increased level of pyruvate in the retinas of pyruvate-supplemented animals (4.891 ± 1.025 ng/μl) when compared to controls (0.615 ± 0.277 ng/μl). The concentration of pyruvate in the supplemented animals equates to approximately 55 μM. These preliminary data attest to the feasibility of the strategy.

**FIGURE 10:**
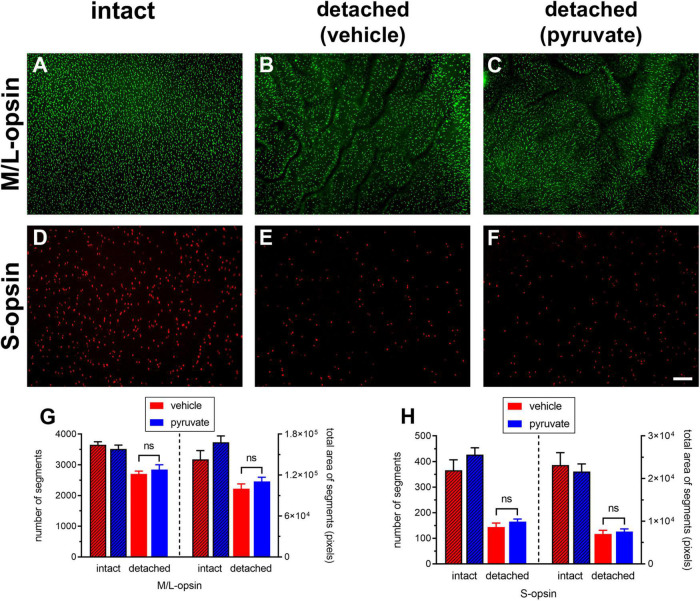
Short-term pyruvate supplementation fails to protect against loss of M/L-opsin^+^ and S-opsin^+^ cone opsins at 3 days after retinal detachment. **(A–F)** Representative wholemount images of M/L-opsin^+^ cones and S-opsin^+^ cones in intact retina **(A,D)**, and at 3 days after detachment in rats administered normal **(B,E)** or pyruvate-supplemented **(C,F)** drinking water **(B,E)**. Scale bar: 100 μm. **(G,H)** Quantification of number and total area of M/L-opsin^+^ and S-opsin^+^ cone segments in vehicle-intact, pyruvate-intact, vehicle-detached, and pyruvate-detached groups after detachment. Values represent mean ± SEM.

**FIGURE 11:**
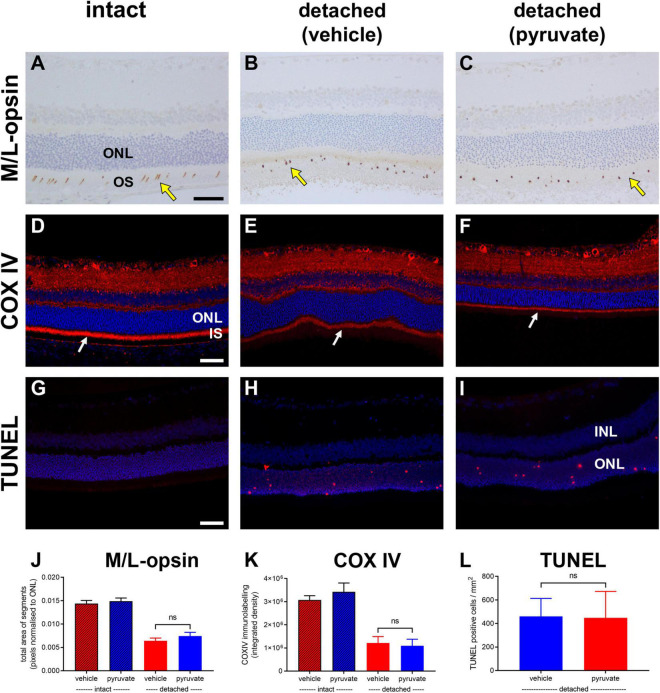
Short-term pyruvate supplementation fails to protect against photoreceptor degenerative changes at 3 days after retinal detachment. **(A–I)** Representative images of M/L-opsin immunolabelling (yellow arrows), COX IV immunolabelling (white arrows), and TUNEL labelling in intact retina **(A,D,G)** and at 3 days after detachment in rats administered normal **(B,E,H)** or pyruvate-supplemented **(C,F,I)** drinking water. Scale bars: 60 μm. INL, inner nuclear layer; IS, inner segments; ONL, outer nuclear layer. **(J–L)** Quantification of total area of M/L-opsin immunolabelling **(J)**, abundance of COX IV immunoreactivity associated with photoreceptor inner segments **(K)**, and the number of TUNEL-positive cells in the ONL **(L)**. All values represent mean ± SEM.

The neuroprotection study comprised two experiments: an early time-point of evaluation that coincides both with the peak of rod apoptosis and the time when cone segment loss becomes evident, and, a late time-point of evaluation when substantial photoreceptor loss has occurred. Initially, we utilised retinal wholemounts and transverse sections to evaluate whether pyruvate administration prevented the early loss of cone opsins that occurs after retinal detachment. Analysis of retinal wholemounts at 3 days after detachment in the vehicle-treated group revealed a modest loss in M/L-opsin^+^ cones (Figures 10A–C,G) relative to intact retinas when evaluated by number of segments (75.5 ± 2.6% remaining) or by total area of segments (63.9 ± 4.6% remaining). Oral supplementation with pyruvate did not significantly preserve M/L-opsin^+^ cones (79.6 ± 4.3% remaining by segment number, *P* = 0.46; 70.7 ± 4.0% remaining by segment area, *P* = 0.29). Consistent with our published findings (Chidlow et al., 2022), there was a much greater susceptibility of S-opsin^+^ cones (Figures 10D–F,H) to retinal detachment relative to intact retinas when evaluated by number of segments (36.1 ± 4.0% remaining) or by total area of segments (32.8 ± 3.9% remaining). Again, pyruvate did not significantly rescue S-opsin^+^ cones (41.5 ± 2.4% remaining by segment number, *P* = 0.27; 35.2 ± 2.7% remaining by segment area, *P* = 0.62). Wholemounts are not an ideal means of quantifying cone segment length, as segments are observed essentially in a two dimensional plane. To shed more light on whether pyruvate preserved cone outer segment morphology, we analysed transverse sections of the retina. At 3 days after detachment there was a clear loss in total area of M/L-opsin^+^ cone segments in the vehicle-treated group when compared to intact retinas (44.6 ± 4.0% remaining; Figures 11A–C,J). As for wholemounts, supplementation with pyruvate did not significantly preserve M/L-opsin^+^ cones (51.7 ± 5.5% remaining; *P* = 0.31). Of note, S-opsin^+^ cones were not quantified in transverse sections as they are sparse in number in the albino rat retina.

Next, we evaluated whether pyruvate administration prevented the early loss of mitochondrial COX IV from photoreceptor inner segments. Analysis of retinal transverse sections at 3 days after detachment in the vehicle-treated group revealed a marked loss in COX IV immunolabeling of inner segments relative to intact retinas (37.6 ± 8.8% remaining; Figures 11D–F,K). Supplementation with pyruvate did not significantly preserve COX IV immunolabeling (33.9 ± 9.4% remaining; *P* = 0.79).

Finally, we evaluated whether pyruvate administration could prevent photoreceptor death using the TUNEL assay, which detects DNA fragmentation in apoptotic or necrotic nuclei. Analysis of retinal transverse sections at 3 days after detachment in the vehicle-treated group revealed 460.6 ± 152.4 cells/mm^2^ in the ONL (Figures 11G–I,L). Administration of pyruvate did not result in significantly fewer TUNEL-positive nuclei within the ONL (447.7 ± 224.5%; *P* = 0.96).

In the second experiment, transverse sections of the retina were analysed 4 weeks after detachment, by which time substantial rod photoreceptor death has occurred. Oral pyruvate was administered throughout the duration of the experiment.

**FIGURE 12:**
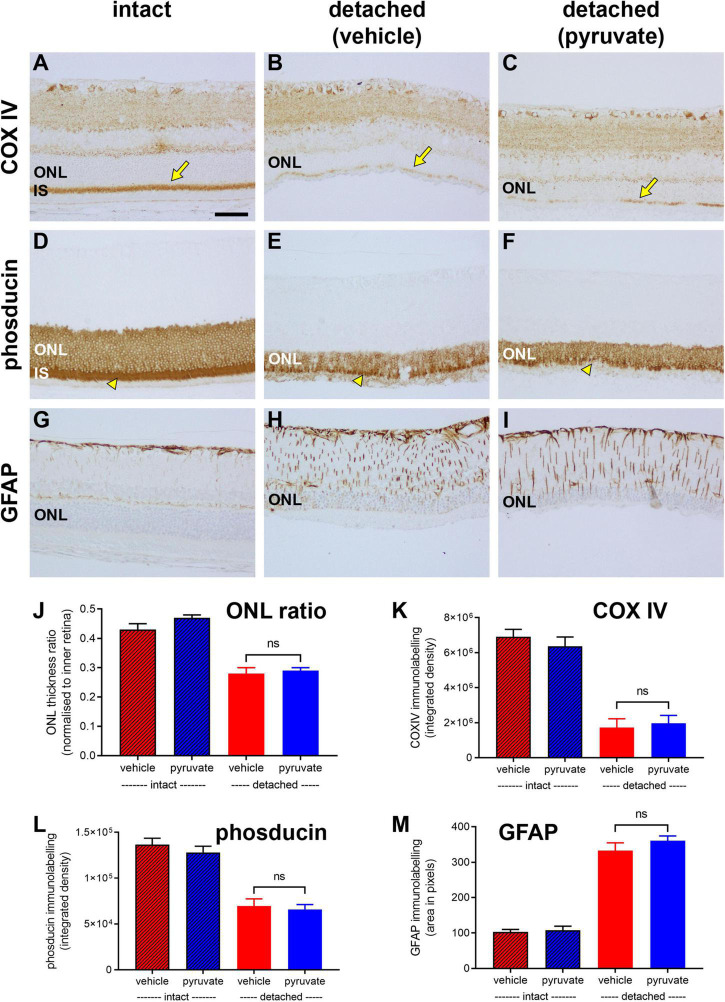
Long-term pyruvate supplementation fails to protect against photoreceptor degenerative changes at 4 weeks after retinal detachment. **(A–I)** Representative images of COX IV (arrows highlight inner segments, IS), phosducin, and GFAP immunoreactivites in intact retina **(A,D,G)**, and at 4 weeks after detachment in rats administered normal **(B,E,H)** or pyruvate-supplemented **(C,F,I)** drinking water. Scale bar: 60 μm. ONL, outer nuclear layer. **(J–M)** Quantification of ONL thickness ratio **(J)**, abundance of COX IV immunoreactivity associated with IS **(K)**, abundance of phosducin immunoreactivity **(L)**, and total area of GFAP immunolabelling **(M)**. All values represent mean ± SEM.

First, we evaluated whether pyruvate administration prevented detachment-induced loss of mitochondrial integrity from photoreceptor inner segments. Analysis of tissue sections revealed a marked loss in COX IV immunolabeling within inner segments in the vehicle-treated group (26.0 ± 7.6% remaining relative to intact retinas; Figures 12A–C,K). Supplementation with pyruvate did not significantly preserve COX IV immunolabeling (29.7 ± 6.8% remaining; *P* = 0.72; Figures 12C,K).

We then determined if pyruvate administration mitigated the loss of phosducin expression by photoreceptors that occurs following prolonged detachment (Chidlow et al., 2022). In intact retinas, phosducin labels rod somas and inner segments (Figure 12D). In the vehicle-treated group, the amount of phosducin signal, evaluated as total integrated density of immunolabeling, had decreased to 52.2 ± 5.9% of the level in intact retinas (Figures 12E,L). Pyruvate did not significantly preserve the level of phosducin (49.4 ± 4.0% remaining; *P* = 0.70; Figures 12F,L).

Next, we evaluated whether pyruvate administration moderated the detachment-induced upregulation of the intermediate filament GFAP. Retinal macroglia are sensitive indicators of tissue injury and have been shown to be greatly affected by prolonged retinal detachment (Verardo et al., 2008). In intact retinas, GFAP immunoreactivity was mainly localised to astrocytes and Müller cell end-feet in the nerve fibre layer (Figure 12G). In the vehicle-treated group, there was a 2.5 ± 0.1 fold upregulation of GFAP, evaluated as total area of immunolabeling, relative to intact retinas (Figures 12H,M). Supplementation with pyruvate did not significantly prevent the GFAP-labelled hypertrophy (29.7 ± 6.8% remaining; *P* = 0.72; Figures 12I,M).

Finally, we assessed cumulative photoreceptor death after detachment by measuring the ratio of ONL to inner retinal thickness (Figure 12J). Analysis of tissue sections revealed a decrease in thickness of the ONL from 0.45 ± 0.1 cells/mm^2^ in intact retinas to 0.28 ± 0.2 cells/mm^2^ in the vehicle-treated group. This loss of photoreceptors was not prevented by supplementation with pyruvate (0.29 ± 0.1 cells/mm^2^, *P* = 0.57).

The overall results of the neuroprotection study indicate that oral pyruvate supplementation failed to prevent rod and cone deconstruction and degeneration after retinal detachment.

## Discussion

The first objective of the present study was to investigate whether experimental detachment in rats causes outer retinal hypoxia. We analysed retinas at 1 day after detachment. There are three reasons for selecting this time point: firstly, if cellular hypoxic responses occur, they would be detectable within 24 h of separation of the tissue from its blood supply, since cells respond rapidly to hypoxia; secondly, photoreceptor deconstruction is at a very early stage at 1 day after detachment (Chidlow et al., 2022). If hypoxia is the principal driver of pathogenesis, it must be present during the early stages of degeneration; thirdly, the detachment is highest at this time, hence photoreceptors are furthest from their blood supply. Examining later time points is essentially redundant, although it is a limitation of this study that hypoxia was only examined at 1 day after detachment. To identify hypoxia in tissue sections, we used both exogenous and endogenous biomarkers, specifically pimonidazole and HIF-1α. Pimonidazole forms stable covalent adducts with proteins in cells with a pO_2_ of less than 10 mmHg (Arteel et al., 1995) and has been widely employed in cancer research, where the amount of pimonidazole that is detected in tumours appears to be proportional to the degree of hypoxia (Bussink et al., 2003). In the retina, pimonidazole has been used to identify hypoxic cells in rodent models of acute ocular hypertension (Holcombe et al., 2008) and oxygen-induced retinopathy (Mowat et al., 2010). We have previously used the methodology to reveal hypoxia within the optic nerve head in rats subjected to experimental glaucoma (Chidlow et al., 2017), and, within the current study, demonstrated positive staining in retinas that had undergone BRVO. Our results showed no unequivocal staining for pimonidazole within any of the six detached retinas analysed, suggesting that the likely reduction in pO_2_ is of insufficient magnitude to produce hypoxia-induced covalent protein adducts.

The negative pimonidazole finding was supported by analysis of HIF-1α expression. The transcription factor HIF-1, a heterodimer comprising α- and β-subunits, is considered the master regulator of cellular responses to hypoxia. Both subunits are constitutively expressed, but the α-subunit alone is rapidly degraded in normoxic conditions (Chua et al., 2010). Cellular hypoxia, i.e., the reduction in available molecular oxygen, reduces the process of HIF-1α degradation, allowing this protein to accumulate and dimerise with the β-subunit to form stabilised HIF-1. HIF-1 then translocates to the nucleus, binds to hypoxia-response elements, and activates a plethora of target genes that facilitate cell adaptation to hypoxia (Lee et al., 2020). We found positive nuclear labelling for HIF-1α in retinas subjected to BRVO, but not in experimentally detached retinas. As for pimonidazole, the data suggest that the likely reduction in local oxygen availability is below the level for stabilisation to occur. An earlier study likewise did not find any HIF-1α nuclear labelling in the ONL after detachment in rats, but did find a positive signal when retinal samples were analysed by Western blotting (Shelby et al., 2015). Finally, we investigated whether there were upregulations of recognised HIF target genes in retinas detached for 1 day. The results showed no consistent pattern of upregulation in a panel of four highly inducible HIF-response genes, although one of the genes, PDK1, was elevated in detached samples, which may be of significance. Nevertheless, the overall data show that, in the rat, experimental detachment appears not to lead to a reduction in pO_2_ within the outer retina that is of sufficient magnitude to produce covalent adducts of pimonidazole, nuclear expression of HIF-1α, or consistent upregulation of HIF target genes.

The combined results suggest that detached photoreceptors do not become hypoxic in an analogous manner to inner retinal cells during BRVO or oxygen-induced retinopathy. In fact, the BRVO retinas not only act as a useful positive control tissue, but inadvertently substantiate our finding that detachment *per se* is not associated with outer retinal hypoxia. The rationale for this declaration is that experimental BRVO (induced by laser occlusion of the retinal vasculature) causes extensive serous retinal detachment after 1 day, yet hypoxia—whether delineated by pimonidazole or HIF-1α–was only observed in the inner retina of BRVO retinas. The lack of measureable hypoxia is arguably surprising given the body of data generated in feline and ground squirrel retinas highlighting the importance of oxygenation for photoreceptor survival. This body of data comprises two components: (1) the use of oxygen microelectrodes together with diffusion modelling has predicted a substantial O_2_ deficit in detached photoreceptors owing to their greater distance from the choroidal vasculature (Linsenmeier and Padnick-Silver, 2000; Wang and Linsenmeier, 2007); (2) hyperoxia therapy in feline (Lewis et al., 1999, 2004; Mervin et al., 1999) and ground squirrel (Sakai et al., 2001) retinas protects against photoreceptor deconstruction and degeneration. The authors of these studies have argued that hyperoxia is protective because it restores oxygen availability and hence allows increased photoreceptor oxygen consumption (Wang and Linsenmeier, 2007). How do we reconcile this apparent discrepancy? Firstly, the situation in rodents may not reflect what happens in cats and ground squirrels: rodent eyes are smaller and hence the height of detachment will be relatively lower, while photoreceptor metabolism may be more glycolytic and less oxidative in rodents than in cats (Chinchore et al., 2017). Indeed, enough oxygen may be supplied by the retinal circulation in rats to circumvent measureable cellular hypoxia; oxygen modelling in cats has shown that the retinal circulation makes a larger contribution to photoreceptor oxygenation in the detached retina than in the attached retina (Wang and Linsenmeier, 2007). Secondly, it needs to be recognised that, to our knowledge, neither pimonidazole nor HIF-1α have been investigated as hypoxia biomarkers in feline or ground squirrel retinas. It is quite possible that photoreceptor oxygen availability, while considerably lower in detached relative to intact cat retina, does not fall to the level required to trigger hypoxic pimonidazole staining. It is also possible that pimonidazole does not diffuse to the detached retina, accounting for the negative findings herein. It would be highly informative to ascertain what degree of protection is afforded to photoreceptors by hyperoxia during retinal detachment in rats.

Equipped with the knowledge that retinal detachment did not cause a reduction in outer retinal pO_2_ that was sufficient to cause pimonidazole staining, HIF1α accumulation or upregulation of HIF target genes, we investigated expression of COX IV in photoreceptor inner segment mitochondria. Interestingly, in this case, our results largely mirrored those reported in feline retina (Mervin et al., 1999; Lewis et al., 2002, 2004), namely minimal alteration to COX IV labelling within the first 24 h of detachment, followed by a striking, sustained loss of COX IV thereafter. These data reveal that detached photoreceptors have a highly compromised capacity to generate ATP from oxidative phosphorylation. However, mitochondria can still generate ATP under low oxygen conditions *via* substrate-level phosphorylation mediated by the enzyme SUCL (Weinberg et al., 2000; Chinopoulos and Seyfried, 2018); thus, we examined if detached photoreceptors continue to express this key matrix enzyme. The data showed SUCL was lost in a similar temporal fashion to COX IV, essentially negating the hypothesis that mitochondrial substrate-level phosphorylation can play any significant role in photoreceptor bioenergetics beyond the first 24 h. When these data are combined with the finding that expression of the mitochondrial antioxidant enzyme SOD2 as well as expression of other intrinsic mitochondrial proteins, such as AGC1 and mAST (enzymes involved in the malate-aspartate shuttle) and mitochondrial creatine kinase were also drastically abrogated during retinal detachment, it must be concluded that detachment likely causes a profound decrease in the number of functional mitochondria in rat photoreceptor inner segments, as has previously been described in feline retina (Fisher et al., 2005).

Loss of mitochondria from inner segments during detachment necessitates that the continuing bioenergetic needs of surviving photoreceptors must be principally met by glycolysis. The switch from oxidative phosphorylation to glycolysis occurs in cells subjected to mitochondrial dysfunction or low oxygen availability—the Pasteur effect—and is typically characterised by upregulated expression of certain glycolytic enzymes such as HK2, PKM2, and LDH-A (Mathupala et al., 2001; Goda and Kanai, 2012). Our data failed to demonstrate any increased expression of glycolytic enzymes during experimental detachment. In fact, there was a generalised downregulation of glycolytic machinery, and a striking loss of HK2, a response also documented by others (Weh et al., 2020; Xiao et al., 2021). The failure to upregulate glycolytic machinery most likely reflects limited availability of glucose. Thus, detached photoreceptors are in an unenviable position: mitochondrial failure means that they cannot utilise other fuels, such as ketone bodies, lactate and amino acids, which hypoglycemic neurons would otherwise direct into the TCA cycle for respiration (Rehni and Dave, 2018). Likewise, there is probably insufficient glucose to increase glycolytic flux to compensate for decreased mitochondrial respiration. Of note, there was a partial recovery of LDH-A, and to a lesser extent GAPDH, in photoreceptors at 4 weeks after detachment. The explanation for this partial recovery is unknown, but may reflect increased oxygenation/nutrient supply due to the generally shallower nature of the detachment at this advanced time point, or it may reflect the fact that many photoreceptors have already been lost, hence there is relatively more oxygen/glucose available to surviving photoreceptors. It is also of interest that detached areas immediately adjacent to intact retina showed higher levels of all bioenergetic proteins investigated. The underlying factor might be related to availability of oxygen or nutrients, or perhaps greater exposure to an RPE-produced survival factor.

The loss of mitochondrial, and downregulation of glycolytic, enzymes during detachment indicates that photoreceptors likely have severely reduced energetic capacity, presumably limiting their metabolic processes to those essential for maintaining homeostasis and ensuring cellular survival. Indeed, it is inevitable that any decrease in the high rate of aerobic glycolysis by photoreceptors will drastically impact upon their high anabolic activity, specifically the diurnal biogenesis of outer segments (Chinchore et al., 2017). In this regard, the role of autophagy in rod and cone deconstruction and survival after retinal detachment appears crucial (Besirli et al., 2011; Chinskey et al., 2014). Autophagy of outer segments will substantially reduce the energetic expenditure of photoreceptors, as well as reducing their need for substrates for aerobic glycolysis. Our findings pose questions such as do detached photoreceptors that have essentially lost inner segment mitochondria retain sufficient energetic capacity to upregulate proteins that may aid their long-term survival? and is inner retinal metabolism altered in detached retinas? In regard to the first question, we showed a delayed, but chronic, upregulation of FGF-2 throughout the ONL following detachment. FGF-2 is an endogenous survival factor that is synthesised by photoreceptors in response to various injuries and which promotes their survival (Wenzel et al., 2005). These data highlight that photoreceptors do not simply “hibernate”; they can still instigate *de novo* synthesis of proteins not normally expressed in the healthy retina, although it is doubtful whether they are able to maintain normal levels of abundant, structurally important proteins, such as proteins involved in visual transduction. Regarding the inner retina, our data suggest that metabolic alterations occur within second order neurons early after detachment. The most striking finding was the immediate downregulation of LDH-A expression by bipolar cells that was evident 1 day after detachment. This was accompanied by reduced expression of GAPDH. It is reasonable to postulate that as visual transduction ceases in photoreceptors, the metabolic workload of bipolar cells reduces dramatically, hence their glycolytic capacity decreases accordingly.

The principal catalyst for photoreceptor degeneration following retinal detachment is presently unknown, but the foremost candidates are hypoxia and hypoglycemia. Our findings that detachment did not cause widespread hypoxia in the outer retina, but did lead to downregulated expression of bioenergetic enzymes by photoreceptors, led us to hypothesise that bioenergetic substrate insufficiency may be critical in terms of pathogenesis, and that boosting metabolic inputs may preserve photoreceptor bioenergetic capacity and prevent their deconstruction and degeneration. To test this hypothesis, we adopted a strategy of oral supplementation with pyruvate, a compound with an excellent safety profile that has recently been used in a phase 2 clinical trial for glaucoma (De Moraes et al., 2022). Pyruvate represents the end-product of aerobic glycolysis and is a major substrate for oxidative metabolism as well as other energy metabolism pathways. It can also be reduced to lactate *via* LDH, thereby regenerating NAD^+^ and affording metabolic flexibility in conditions of bioenergetic stress (Olson et al., 2016). Furthermore, pyruvate displays antioxidant properties, and has been shown to protect the retina against oxidative stress (Hegde and Varma, 2008; Hegde et al., 2010). Deletion of the mitochondrial pyruvate carrier MPC1 causes a retinal mitochondrial energy deficit and impairs rod and cone function, attesting to the role that pyruvate plays in photoreceptor homeostasis (Grenell et al., 2019). Prior to testing the effect of pyruvate in the detachment model, we evaluated whether pyruvate prevented aglycemia-induced photoreceptor loss in mixed retinal cultures. The data showed a strong protective effect of pyruvate over a large concentration range, although lower doses did not provide complete protection. The results are in agreement with earlier studies showing that pyruvate counteracted neuronal death induced by hypoglycemia in the hippocampus and cerebral cortex (Izumi et al., 1994; Julio-Amilpas et al., 2015). Next, we assessed its retinal bioavailability after 2 weeks of oral supplementation in healthy rats. The results showed an 8-fold elevation in retinal pyruvate. Despite these auspicious preliminary data, we obtained comprehensively negative results when analysing the efficacy of pyruvate in protecting against photoreceptor injury caused by retinal detachment. Chronic pyruvate supplementation neither protected against apoptotic death of rods, nor early losses of cone opsins, nor COX IV-labelled mitochondria when evaluated at 3 days after detachment. The regimen was also ineffective against cumulative photoreceptor deconstruction and degeneration when evaluated after 4 weeks. The lack of neuroprotective efficacy may be related to pyruvate bioavailability, despite the fact that an identical treatment regimen afforded robust protection to retinal ganglion cells in rat and mouse models of glaucoma (Harder et al., 2020). It is a limitation that we did not measure pyruvate bioavailability after 1 week, the timepoint of experimental detachment. It is conceivable that the pyruvate level was lower at 1 week than when measured after 2 weeks of administration. Furthermore, pyruvate does still need to reach detached photoreceptors from the vasculature, which is less straightforward than for glaucoma, although the inner retinal blood supply, in addition to the choroid, would be a potential source of pyruvate. It also needs to be recognised that the supplementation regime only increased retinal pyruvate bioavailability by approximately 50 μM, a concentration that does not fully protect photoreceptors in culture from nutrient deprivation.

An alternative explanation for the failure of pyruvate to sustain photoreceptors in the detached environment is that the steady state pO_2_ is inadequate for photoreceptors to operate oxidative phosphorylation to a level sufficient to prevent photoreceptor inner segment deconstruction and loss of mitochondria. This is a reasonable assumption as photoreceptors have an extremely high metabolic rate, yet a limited mitochondrial reserve capacity (Kooragayala et al., 2015). Any reduction in oxygen availability will have a detrimental impact upon ATP production and cellular homeostasis. Nevertheless, even with insufficient oxygen, supplemental pyruvate ought to provide a suitable carbon source for mitochondrial substrate-level phosphorylation. There are a wealth of studies showing that regimens boosting substrate-level phosphorylation support metabolism and cellular survival under conditions of anoxia (Chinopoulos, 2013), although many of these studies employed the citric acid intermediate α-ketoglutarate. It is possible that MPC1 or pyruvate dehydrogenase—which has a requirement for NAD^+^ that is normally replenished by the electron transport chain—do not operate efficiently within the detached environment, and this prevented exogenous pyruvate from fuelling mitochondrial ATP production. Aspartate or α-ketoglutarate may prove more suitable fuels for substrate-level phosphorylation. Indeed, oral supplementation with α-ketoglutarate has recently been shown to protect photoreceptors in the Pde6 mouse model of retinitis pigmentosa (Rowe et al., 2021), which provides support for this hypothesis. Future studies could investigate the neuroprotective efficacy of α-ketoglutarate in experimental retinal detachment. In considering our results, it also needs to be recognised that healthy photoreceptors are believed to have an aerobic glycolytic profile, with only a small proportion of pyruvate generated by glycolysis being oxidised within mitochondria (Hurley, 2021). Indeed, MPC1 deletion does not actually cause photoreceptor degeneration, impacting the inner retina to a greater extent than the outer retina (Grenell et al., 2019). It may be the case that boosting glycolysis, by chronically elevating the glucose concentration, would protect detached photoreceptors. This strategy, which on a practical basis involves rendering animals diabetic, was not considered for ethical and translational reasons, but would be interesting from a mechanistic perspective. Indeed, there is evidence from animal models of retinitis pigmentosa that chronic enhancement of glycolysis promotes rod and cone survival (Ait-Ali et al., 2015; Zhang et al., 2016).

One final hypothesis that might explain the failure of pyruvate to protect photoreceptors during retinal detachment is that the pathogenesis is not driven by bioenergetic failure *per se*, but by neuroinflammation caused either by physical disruption and separation of the photoreceptors from the RPE, or by altered oxygen/nutrient availability. Neuroinflammation is believed to play a key role in many degenerative retinal diseases, including inherited retinal dystrophies and age-related macular degeneration (Madeira et al., 2015; Olivares-Gonzalez et al., 2021). While such data are outside the scope of this manuscript, it is relevant that we measured a marked upregulation of one of the quintessential drivers of neuroinflammation, TNFα, in 1 day detached samples analysed by qPCR. The role of microglia-derived neuroinflammation in retinal detachment needs further study.

Retinal metabolism, particularly the bioenergetic profiles and pathological responses of the various cellular subtypes still presents a considerable knowledge gap that has important clinical consequences. The current study perhaps raises more questions than it answers, but provides a foundation and motivation for future research in this area. It is, however, important to caution that the data presented within this manuscript pertaining to the bioenergetic status of the retina in general, and the photoreceptors in particular, are specific to experimental retinal detachment, and it would be unwise to draw definitive conclusions about the “normal” physiological state of retinal cells.

## Data availability statement

The raw data supporting the conclusions of this article will be made available by the authors, without undue reservation.

## Ethics statement

This animal study was reviewed and approved by Animal Ethics Committee, University of Adelaide (Adelaide, Australia).

## Author contributions

GC, JW, and RC: study concept and design. WC: establishment of rat model of retinal detachment. GC and JW: acquisition of data and analysis and interpretation of data. GC: drafting of the manuscript. JW, WC, and RC: critical revision of the manuscript for important intellectual content. GC and RC: statistical analysis. RC and WC: obtain funding. RC: administrative, technical, and material support. All authors had full access to all the data in the study and took responsibility for the integrity of the data and the accuracy of the data analysis.

## Supplementary material

Supplementary Figure 1 Double labelling immunofluorescence of LDH-A with the ON-bipolar cell marker PKCα. LDH-A [**(A)** green, arrow] colocalises with PKCα [**(B)** red, arrow] as seen in [**(C)** arrow] the merged image. Scale bar: 30 μm; GCL, ganglion cell layer; INL, inner nuclear layer; ONL, outer nuclear layer.
