# Supplementary material for: Investigations into photoreceptor energy metabolism during experimental retinal detachment
Source: Front Cell Neurosci. 2022 Nov 18;16:1036834. doi: 10.3389/fncel.2022.1036834 (PMC9716104; doi:10.3389/fncel.2022.1036834)
Supplement: Supplementary file 1 [file Data_Sheet_1.docx]

**Supplementary Table 1. Study Design**

| **Characterization Study** | | | |
| --- | --- | --- | --- |
| *Time point* | *Analysis* | *n* | *notes* |
| day 1 | immunohistochemistry | 9 | n=6 of which received pimonidazole |
| day 1 | COX IV enzyme activity | 3 |  |
| day 1 | qPCR | 7 | retinas dissected into intact and detached portions |
| day 3 | immunohistochemistry | 10 |  |
| day 7 | COX IV enzyme activity | 3 |  |
| day 7 | immunohistochemistry | 8 |  |
| day 28 | immunohistochemistry | 12 |  |
| **Neuroprotection Study** | | | |
| *Time point* | *Analysis* | *n* | *notes* |
| day 3 | wholemounts | 7 | per group (vehicle or pyruvate) |
| day 3 | immunohistochemistry | 7-10 | per group (vehicle or pyruvate) |
| day 28 | immunohistochemistry | 13-15 | per group (vehicle or pyruvate) |

**Supplementary Table 2. Primer sequences for mRNAs amplified by real-time RT-PCR**

| **mRNA** | **Primer sequences** | **Product** | **annealing temperature** | **Accession**  **number** |
| --- | --- | --- | --- | --- |
| ADM | 5’-GTTTCCATCGCCCTGATGTTATTG-3’  5’-GCTGCTGGACGCTTGTAGTTC-3’ | 144 | 63ºC | NM_012715 |
| BNIP3 | 5’-ATGGGATTGGTCAAGTCGGC-3’  5’-CTTCCAATGTAGATCCCCAAT-3’ | 205 | 61°C | NM_053420 |
| cyclophilin | 5’-GTGTTCTTCGACATCACGGCT-3’  5’-CTGTCTTTGGAACTTTGTCTGCA-3’ | 82 | 63º C | NM_017101 |
| HK2 | 5’-AAAGAGAACAAGGGCGAGGAG-3’  5’-GAGGAAGCGGACATCACAGTC-3’ | 138 | 63 °C | NM_012735 |
| HPRT1 | 5’-GTCATCAGCGAAAGTGGAAAAG-3’  5’-ATCAAAAGGGACGCAGCAAC-3’ | 206 | 61°C | NM_012583 |
| OPN1MW | 5’-CATCCGAGCAGTGGCAAAG-3’  5’-CACAAGAGGGTGGAAGGCATAG-3’ | 175 | 63ºC | NM_053548 |
| PDK1 | 5’-GTTCCGTCCCATCTCTATCACA-3’  5’-GAACTTGAATCGGGGGATAAACG-3’ | 106 | 61°C | NM_053826 |
| SLC2A1 | 5’-TCCACCACACTCACCACACTC-3’  5’-CATAAGCACGGCAGACACAAA-3’ | 159 | 63ºC | NM_138827 |
| TNFα | 5’-AAATGGGCTCCCTCTCATCAGTTC-3’  5’-TCTGCTTGGTGGTTTGCTACGAC-3’ | 111 | 64ºC | NM_012675 |

ADM, adrenomedullin; BCL2 interacting protein 3, BNIP3; HK2, hexokinase 2; PDK1, pyruvate dehydrogenase kinase 1
