# Supplementary figures and images for: Investigations into photoreceptor energy metabolism during experimental retinal detachment
Source: Front Cell Neurosci. 2022 Nov 18;16:1036834. doi: 10.3389/fncel.2022.1036834 (PMC9716104; doi:10.3389/fncel.2022.1036834)

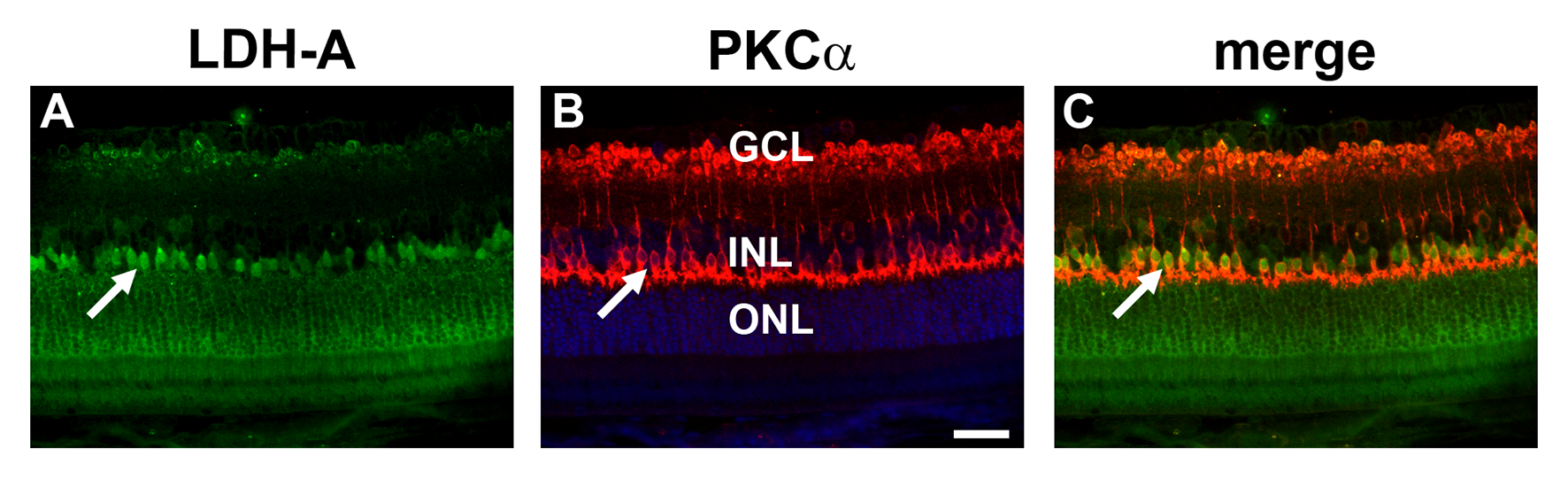

Supplement: Supplementary Figure 1 — Double labelling immunofluorescence of LDH-A with the ON-bipolar cell marker PKCα. LDH-A [(A) green, arrow] colocalises with PKCα [(B) red, arrow] as seen in [(C) arrow] the merged image. Scale bar: 30 μm; GCL, ganglion cell layer; INL, inner nuclear layer; ONL, outer nuclear layer. [file Image_1.TIF]
